# Why Worry about How Many Species and Their Loss?

**DOI:** 10.1371/journal.pbio.1001130

**Published:** 2011-08-23

**Authors:** Robert M. May

**Affiliations:** Zoology Department, Oxford University, Oxford, United Kingdom

## Abstract

We are astonishingly ignorant about how many species are alive on earth today, and even more ignorant about how many we can lose yet still maintain ecosystem services that humanity ultimately depends upon. Mora et al.'s paper is important in offering an imaginative new approach to assessing total species numbers, both on land and in the sea.

It is a remarkable testament to humanity's narcissism that we know the number of books in the US Library of Congress on 1 February 2011 was 22,194,656, but cannot tell you—to within an order-of-magnitude—how many distinct species of plants and animals we share our world with [1]. Something like 1.5 million distinct eukaryotes have been named and recorded, but, lacking synoptic databases, even this number is uncertain owing to synonyms (the same species separately named in two or more different collections) [2].

Part of the problem is that taxonomic effort is approximately divided 1∶ 1∶ 1 among vertebrates, plants, and invertebrates, whereas plant species are roughly 10 times, and invertebrates 100 times, more numerous than vertebrates [3]. Mammals and birds are the best known, again reflecting our narcissism: their features are akin to our own.

In this issue of *PLoS Biology*, Mora et al. [4] offer an interesting new approach to estimating the total number of distinct eukaryotic species alive on earth today. They begin with an excellent survey of the wide variety of previous estimates, which give a range of different numbers in the broad interval 3 to 100 million species. I have favoured a number between 2 and 10 million, and if I had to buy a ticket in a sweepstakes, I'd have chosen 5 million.

Mora et al.'s imaginative new approach begins by looking at the hierarchy of taxonomic categories, from the details of species and genera, through orders and classes, to phyla and kingdoms. They documented the fact that for eukaryotes, the higher taxonomic categories are “much more completely described than lower levels”, which in retrospect is perhaps not surprising. They also show that, within well-known taxonomic groups, the relative numbers of species assigned to phylum, class, order, family, genus, and species follow consistent patterns. If one assumes these predictable patterns also hold for less well-studied groups, the more secure information about phyla and class can be used to estimate the total number of distinct species within a given group.

In this way, Mora et al. arrive at a global total of 8.7 million eukaryotic species, with a standard error of ±1.3 million. Most are terrestrial, with 2.2 (±0.2) million being marine.

This is higher than my earlier “best guess”, but I like the simplicity of this new method.

Currently, diligent field taxonomists are adding newly discovered species at the rate of very roughly 15,000 each year (when discounted for synonyms) [2]. Given that we currently recognize something like 1.5 million distinct eukaryotic species, Mora et al.'s estimated species number suggests 480 years to finish the job. It is, however, reasonable to expect that in the near future, molecular methods—“barcode taxonomy”—will greatly speed up the task of keying-out collected material, as well as resolving synonymies [5]. But the basic field activity of collecting new material will remain a rate-limiting step. Increasing the number of people engaged on the task would obviously help, and any such increase could be made more effective—as pioneering efforts in Costa Rica and elsewhere have shown—by using “parataxonomists”, local people who use rough morphological criteria to help recognise new species, in combination with taxonomic experts. All in all, my optimistic guess would be around a century to complete our assessment of the diversity of life on earth.

But this tentative assessment makes no allowance for accelerating extinction rates. As the Millennium Ecosystem Assessment emphasized [6], over the past century documented extinctions within well-studied groups (particularly birds and mammals) were at a rate 10^3±1^ higher than the average extinction rate seen over the half-billion-year sweep of the fossil record [7],[8]. One can draw no comfort from the thought that the task of cataloguing our planet's biological richness will be simplified by its winnowing.

Ultimately, why should we care about how many species are alive on earth today, and about how many of them are known to us? One notable Victorian physicist (I will be merciful and not name him) opined that such a quest is little more than stamp collecting. To the contrary, we increasingly recognise that such knowledge is important for full understanding of the ecological and evolutionary processes which created, and which are struggling to maintain, the diverse biological riches we are heir to. Such biodiversity is much more than beauty and wonder, important though that is. It also underpins ecosystem services that—although not counted in conventional GDP—humanity is dependent upon.

Turning from the general to the specific, I give just one among a multitude of possible concrete examples of beneficial application of taxonomic discovery. In the 1970s Yuan Longping, “the father of rice”, discovered in the wild a new variety of rice, whose cross with a conventional strain led to a new variety that is 30% more efficient. This has motivated subsequent initiatives to document and protect all wild varieties of rice, which obviously can only be done if we have the appropriate taxonomic knowledge. Given the looming problems of feeding a still-growing world population, the potential benefits of ramping up such exploration are clear.

The essential fact is that, if we are to meet the challenges facing tomorrow's world, we need a clearer understanding of how many species there are—both on land and in the even less well-studied oceans—underpinning the structure and functioning of ecosystems. Mora et al.'s interesting new approach to assessing the magnitude of this task is thus very helpful.

## References

[1] May R. M, Raven P. H (1997). The dimensions of life on Earth.. Nature and human society.

[2] Solow R. R, Mound L. A, Gaston K. J (1995). Estimating the rate of synonymy.. Syst Biol.

[3] Gaston K. J, May R. M (1992). The taxonomy of taxonomists.. Nature.

[4] Mora C, Tittensor D. P, Adl S, Simpson A. G. B, Worm B (2011). How many species are there on Earth and in the ocean?. PLoS Biol.

[5] Godfray H. C. J, Knapp S (2004). Introduction: taxonomy for the twenty-first century.. Philos Trans R Soc Lond B Biol Sci.

[6] Millennium Ecosystem Assessment (MEA) (2005). Ecosystems and human well-being: synthesis.

[7] May R. M, Lawton J. H, Stork N. E, Lawton J. H, May R. M (1995). Assessing extinction rates.. Extinction rates.

[8] Pimm S. L, Brooks T. M, Raven P. H (2005). The sixth extinction: how large, where, and when?. Nature and human society.

